# Functional brain network alterations in a rat model of dental malocclusion

**DOI:** 10.3389/fnins.2026.1873417

**Published:** 2026-07-28

**Authors:** Qian Lv, Jiali Xu, Yuejiao Zhang, Shuang Chen, Meiqing Wang

**Affiliations:** 1 State Key Laboratory of Brain Function and Disorders and MOE Frontiers Center for Brain Science and the Institutes of Brain Science, Fudan University, Shanghai, China; 2 Department of Oral Anatomy and Physiology, Shanghai Stomatological Hospital and School of Stomatology, Fudan University, Shanghai, China; 3 Department of Prosthodontics, Shanghai Stomatological Hospital and School of Stomatology, Fudan University, Shanghai, China; 4 Department of Oral Anatomy and Physiology, College of Stomatology, The Fourth Military Medical University, Xi’an, China

**Keywords:** amygdala, anxiety, brain network, dental malocclusion, functional MRI, orofacial proprioceptive pathway

## Abstract

**Background:**

Malocclusion has been associated with alterations in central nervous system function; however, the effects of persistent abnormal occlusal input on whole-brain functional organization remain poorly understood. This study aimed to investigate functional brain network reorganization associated with unilateral anterior crossbite (UAC) in rats.

**Methods:**

Fourteen female rats were used, seven of which were treated with left-sided UAC at 6 weeks old and the other seven served as sham-operation controls. Resting-state functional magnetic resonance imaging data were acquired at 26 weeks old. Regional activity in the proprioceptive pathway was assessed, followed by graph theoretical analysis of whole-brain functional networks.

**Results:**

UAC rats exhibited altered regional activity in the right ventral posteromedial thalamus (*p* = 0.023) and primary somatosensory cortex (*p* < 0.001), contralateral to the modeling side. Network analysis revealed significantly higher normalized clustering coefficient (*p* = 0.014) and small-worldness (*p* = 0.006) in the UAC group compared with controls. Furthermore, UAC rats showed enhanced functional connectivity within a subnetwork (*p* = 0.023) in which the right amygdala exhibited the greatest number of altered connections. In addition, abnormal regional activity was also detected in the right amygdala of UAC rats (*p* = 0.039).

**Conclusion:**

Long-term UAC in rats is associated with alterations in whole-brain functional network organization. These findings provide new insights into the central neural responses associated with abnormal occlusal input.

## 1 Introduction

Malocclusion, characterized by an abnormal alignment or occlusal relationship between the maxillary and mandibular dentition, is one of the most prevalent oral conditions worldwide (Zou et al., 2018; Cenzato et al., 2021). In addition to its well-recognized associations with impaired mastication, temporomandibular joint dysfunction, and craniofacial developmental abnormalities, increasing evidence suggests that malocclusion exerts broader physiological effects beyond the stomatognathic system, including influences on central nervous system (Ono et al., 2010). Clinical studies have reported that patients with occlusal abnormalities and temporomandibular disorders (TMD) frequently exhibit alterations in sensory processing, emotional well-being, and brain function (Reis et al., 2022; Alsulaiman et al., 2025; Wan et al., 2025). Neuroimaging studies have further demonstrated abnormal brain activity and functional connectivity in sensorimotor, limbic, and default mode networks in patients with TMD (Yin et al., 2020; Shrivastava and Ye, 2023). However, because clinical observations are often confounded by chronic pain, emotional disturbances, medication use, and disease duration, the causal effects of abnormal occlusal input on brain function remain poorly understood.

Animal models provide a valuable platform for investigating the neural consequences of chronic malocclusion under controlled experimental conditions. Our previous study (Zhang et al., 2013) established a unilateral anterior crossbite (UAC) model by bonding a metal tube to the left maxillary and mandibular incisors and creating a crossbite relation. This model reproduces key features of chronic malocclusion and has been widely used to investigate temporomandibular joint degeneration and central nervous system alterations induced by abnormal occlusal loading. Our previous studies have demonstrated that UAC induces widespread neural adaptations and behavioral changes (Liu et al., 2019; Ji et al., 2022). Although ipsilateral orofacial hyperalgesia is observed in UAC rats, the pressure pain threshold returns to baseline within 2 weeks (Jing et al., 2017), suggesting that long-term neural alterations are unlikely to be explained solely by persistent nociceptive input. Occlusal information is continuously transmitted to the brain through trigeminal sensory and proprioceptive pathways. In a recent study, we identified a neural pathway extending from the mesencephalic trigeminal nucleus (Vme) to the principal sensory trigeminal nucleus (Vpdm), then to the ventral posteromedial nucleus of the thalamus (VPM), and finally to the primary somatosensory cortex (S1), which conveys orofacial proprioceptive information to the cortex (Ji et al., 2022). Obviously, the altered occlusion leads to an altered activity within the trigeminal-thalamic-cortical circuit in the central network. While previous studies have primarily focused on individual brain regions or specific neural pathways, brain function is increasingly recognized as emerging from complex interactions among distributed neural systems (Bassett and Sporns, 2017). The brain operates as a large-scale network in which multiple regions dynamically interact to support sensory processing, cognition, and emotional regulation. It is highly possible that persistent disturbances in occlusal proprioceptive input would induce neural adaptations beyond primary sensory regions, and UAC could be associated with large-scale reorganization of functional brain networks, yet remains undetermined.

Resting-state functional magnetic resonance imaging (rs-fMRI) has become a widely used technique for constructing whole-brain functional networks in both rodents and humans (van den Heuvel and Hulshoff Pol, 2010; Gozzi and Zerbi, 2023). This approach noninvasively measures spontaneous fluctuations in blood oxygen level-dependent (BOLD) signals across the brain, reflecting intrinsic neural activity in the absence of explicit tasks (Fox and Raichle, 2007). Combined with graph theoretical analysis, rs-fMRI enables quantitative assessment of network topology and identification of key regions that contribute to large-scale neural communication. Importantly, brain networks in rodents and humans share conserved organizational properties, allowing findings from rodent models to provide translational insights into human brain function (Pagani et al., 2023). Although neuroimaging studies have revealed functional network abnormalities in patients with TMD, whether chronic abnormal occlusal input itself is sufficient to induce whole-brain network reorganization is worth of investigation.

In the present study, we applied rs-fMRI and graph theoretical analysis to quantitatively investigate whole-brain functional network organization in rats subjected to long-term UAC. In addition, we examined neural activity in the VPM-S1 pathway, a major downstream component of the trigeminal proprioceptive system that conveys abnormal occlusal information to the cortex. By integrating regional activity analysis with whole-brain network characterization, we aimed to investigate potential alterations in brain functional organization associated with chronic malocclusion and to identify brain regions showing concomitant changes. Together, these analyses may provide preliminary insights into brain functional alterations associated with abnormal occlusal input and help bridge circuit-level mechanisms with large-scale brain network reorganization.

## 2 Materials and methods

### 2.1 Animals

Fourteen 6-week-old female Sprague–Dawley rats were obtained from the Animal Center of the Fourth Military Medical University. The animals were randomly assigned to either the UAC group or the control group (*n* = 7 per group). All rats were housed in a temperature-controlled environment (23 ± 2 °C) under a 12-h light/12-h dark cycle. Standard laboratory chow and bottled water were provided *ad libitum*. All experimental procedures and animal care protocols were approved by the University Ethics Committee and conducted in accordance with institutional guidelines (2023-KQ-052).

### 2.2 UAC model

The UAC model was established following the procedure described in our previous study (Zhang et al., 2013). Briefly, rats were anesthetized with a single intraperitoneal injection of 1% pentobarbital sodium (0.35 mL/100 g). A small metal tube (length = 2.5 mm, inner diameter = 3 mm) was bonded to the left maxillary incisor, while another metal tube (length = 4.5 mm, inner diameter = 3.5 mm) was bonded to the left mandibular incisor. The end of the mandibular tube was bent to form a 135° angle toward the labial side to create a crossbite relationship between the upper and lower incisors. Each procedure was completed within 5 min, and all efforts were made to minimize animal discomfort. No detachment of the metal tubes was observed throughout the experimental period. Control rats underwent the same procedure, except that no metal tubes were attached.

### 2.3 MRI data acquisition

MRI scanning was performed 20 weeks after UAC modeling. To minimize the metal artifacts, the metal tubes were removed 1 day prior to MRI scanning. MRI scans were conducted using a 9.4 T/30 cm uMR (United Imaging Life Science Instrument, Wuhan, China) equipped with an 86 mm diameter transmit volume coil and a single-loop receive coil designed for rats.

Animal preparation during MRI was performed according to previous studies (Brynildsen et al., 2017; Zhu et al., 2024). Rats were initially anesthetized with 3.5% isoflurane, and a bolus of dexmedetomidine (0.01 mg/kg) was administered intraperitoneally. The animals were then positioned in the cradle, and a continuous subcutaneous infusion of dexmedetomidine (0.03 mg/kg/h) was maintained throughout the scan. Isoflurane was delivered at 1.5–2% during preparation and gradually reduced to around 0.5% during scanning to maintain stable physiological conditions. Respiration and body temperature (SA Instruments, Stony Brook, NY, USA) were continuously monitored, and body temperature was maintained between 36.5 and 37.5 °C.

T2-weighted structural images were acquired using a fast spin-echo sequence with the following parameters: echo time (TE) = 47 ms; repetition time (TR) = 3,238 ms; matrix size = 416 × 416; field of view (FOV) = 35 × 35 mm; 30 coronal slices with a thickness of 1 mm; voxel size = 0.084 × 0.084 × 1 mm; echo train length = 13; 4 averages. Resting-state fMRI data were acquired using a single-shot gradient-echo echo-planar imaging (EPI) sequence with the following parameters: TR = 2,000 ms; TE = 15 ms; FOV = 26 × 26 mm; acquisition matrix = 72 × 72; 22 slices with a thickness of 1 mm; voxel size = 0.36 × 0.36 × 1 mm; 300 time points.

### 2.4 MRI data preprocessing

Preprocessing of the fMRI data were performed using the open-source RABIES software^1^ (Desrosiers-Gregoire et al., 2024). Temporal spikes were first corrected for at each voxel. The head motion parameters are estimated by realigning each EPI frame to the mean EPI images using rigid-body registration. Structural images were corrected for intensity inhomogeneities and iteratively registered to generate an unbiased data-driven template. This template was subsequently nonlinearly registered to the SIGMA rat brain template (Barriere et al., 2019). To correct for EPI susceptibility distortions, volumetric EPI images were also corrected for inhomogeneity and nonlinearly registered to the anatomical scan from the same session. After calculating the transformations required to correct for head motion and susceptibility distortions, slice-timing correction was applied to the time series. All transformations were concatenated into a single resampling step applied to each EPI frame, generating preprocessed time series in native space. Data were also resampled into common atlas space at a voxel resolution of 0.4 × 0.4 × 0.4 mm.

Confound regression was performed on time series resampled in common space. First-order detrending was applied to remove linear drifts and the mean signal. Band-pass filtering (0.01–0.1 Hz) was then applied, and 30 s at both ends of the time series were discarded to minimize edge artifacts. The six rigid-body motion parameters and mean signals from white matter and cerebrospinal fluid were regressed out. Finally, spatial smoothing was applied using a Gaussian kernel with a full-width at half-maximum of 1.0 mm.

### 2.5 Regional metric analysis

Regional brain activity was quantified using regional homogeneity (ReHo) and amplitude of low-frequency fluctuation (ALFF) implemented in the DPARSF toolbox (Chao-Gan and Yu-Feng, 2010). ReHo was calculated as Kendall’s coefficient of concordance among a given voxel and its 27 neighboring voxels, reflecting local synchronization of BOLD time series. ReHo calculation was performed prior to spatial smoothing. ALFF reflects the amplitude of spontaneous low-frequency BOLD fluctuations. For ALFF calculation, unfiltered fMRI data were transformed into the frequency domain using a fast Fourier transform. The averaged power spectrum within the frequency range of 0.01–0.1 Hz was calculated for each voxel. For both ALFF and ReHo, subject-level maps were standardized into Z-score maps. Mean values within predefined regions of interest (ROIs) were extracted for later analysis. Bilateral VPM and S1 were defined according to the SIGMA rat brain atlas.

### 2.6 Brain network construction and analysis

Brain network construction and graph theoretical analysis were performed following our previous study (Lv et al., 2016; Yin et al., 2019; Chen et al., 2022) with the GRETNA toolbox (Wang et al., 2015). An atlas consisting of 88 regions of interest was used to parcellate the rat brain based on Schwarz’s atlas (Table 1) (Schwarz et al., 2006). The mean time series within each ROI was extracted, and Pearson correlation coefficients were calculated between all pairs of ROIs to generate whole-brain functional connectivity matrices. A sparsity threshold was then applied to the absolute functional connectivity matrices to generate binary networks. For each correlation matrix, the threshold range was determined according to two criteria: (1) the average degree of the thresholded network was greater than 2 × log(*N*), where *N* = 88; and (2) the resulting networks exhibited small-world properties, with a normalized clustering coefficient (*γ*) greater than 1 and a normalized characteristic path length (*λ*) approximately equal to 1 (Watts and Strogatz, 1998; Achard and Bullmore, 2007). Network analyses were subsequently performed over a sparsity range of 0.05–0.30 with an interval of 0.02. Global network properties were calculated, including small-world parameters (normalized clustering coefficient γ, normalized characteristic path length λ, and small-worldness *σ*) as well as network efficiency measures (global efficiency and local efficiency). For calculation of γ and λ, 100 random networks were generated for each binary network. To obtain a summary metric independent of threshold selection, the area under the curve (AUC) across the entire sparsity range was calculated for each network metric.

**Table 1:** Brain atlas used in the study.

Index	Region name	Abbreviations	Group
1	Cerebellum_Left	lCB	Isocortex
2	Cerebellum_Right	rCB	Isocortex
3	Cortex_Auditory_Left	lAud	Isocortex
4	Cortex_Auditory_Right	rAud	Isocortex
5	Cortex_Cingulate_Left	lCA	Isocortex
6	Cortex_Cingulate_Right	rCA	Isocortex
7	Cortex_Entorhinal_Left	lEnt	Isocortex
8	Cortex_Entorhinal_Right	rEnt	Isocortex
9	Cortex_Frontal_Association_Left	lFRA	Isocortex
10	Cortex_Frontal_Association_Right	rFRA	Isocortex
11	Cortex_Insular_Left	lIns	Isocortex
12	Cortex_Insular_Right	rIns	Isocortex
13	Cortex_MedialPrefrontal_Left	lMPFC	Isocortex
14	Cortex_MedialPrefrontal_Right	rMPFC	Isocortex
15	Cortex_Motor_Left	lMC	Isocortex
16	Cortex_Motor_Right	rMC	Isocortex
17	Cortex_Orbitofrontal_Left	lOFC	Isocortex
18	Cortex_Orbitofrontal_Right	rOFC	Isocortex
19	Cortex_Parietal_Association_Left	lPAC	Isocortex
20	Cortex_Parietal_Association_Right	rPAC	Isocortex
21	Cortex_Piriform_Left	lPIR	Isocortex
22	Cortex_Piriform_Right	rPIR	Isocortex
23	Cortex_Retrosplenial_Left	lRSC	Isocortex
24	Cortex_Retrosplenial_Right	rRSC	Isocortex
25	Cortex_Somatosensory_Left	lS1	Isocortex
26	Cortex_Somatosensory_Right	rS1	Isocortex
27	Cortex_TemporalAssociation_Left	lTAC	Isocortex
28	Cortex_TemporalAssociation_Right	rTAC	Isocortex
29	Cortex_Visual_Left	lV1	Isocortex
30	Cortex_Visual_Right	rV1	Hipp/Amy
31	Hippocampus_AnteroDorsal_Left	lHIPad	Hipp/Amy
32	Hippocampus_AnteroDorsal_Right	rHIPad	Hipp/Amy
33	Hippocampus_Posterior_Left	lHIPp	Hipp/Amy
34	Hippocampus_Posterior_Right	rHIPp	Hipp/Amy
35	Hippocampus_PosteroDorsal_Left	lHIPpd	Hipp/Amy
36	Hippocampus_PosteroDorsal_Right	rHIPpd	Hipp/Amy
37	Hippocampus_Subiculum_Left	lHIPsub	Hipp/Amy
38	Hippocampus_Subiculum_Right	rHIPsub	Hipp/Amy
39	Hippocampus_Ventral_Left	lHIPv	Hipp/Amy
40	Hippocampus_Ventral_Right	rHIPv	Hipp/Amy
41	Amygdala_Left	lAmy	Hipp/Amy
42	Amygdala_Right	rAmy	Hipp/Amy
43	Accumbens_Core_Left	lAcbc	Striatum
44	Accumbens_Core_Right	rAcbc	Striatum
45	Accumbens_Shell_Left	lAcbs	Striatum
46	AccumbensShell_Right	rAcbs	Striatum
47	Caudate_Putamen_Left	lCPu	Striatum
48	CaudatePutamen_Right	rCPu	Striatum
49	Globus_Pallidus_Left	lPAL	Striatum
50	Globus_Pallidus_Right	rPAL	Striatum
51	Ventral_Pallidum_Left	lVP	Striatum
52	Ventral_Pallidum_Right	rVP	Striatum
53	Thalamus_Dorsolateral_Left	lThAdl	Tha/HY
54	Thalamus_Dorsolateral_Right	rThAdl	Tha/HY
55	Thalamus_MidlineDorsal_Left	lTHAmd	Tha/HY
56	Thalamus_MidlineDorsal_Right	rTHAmd	Tha/HY
57	Thalamus_Ventromedial_Left	lTHAvm	Tha/HY
58	Thalamus_Ventromedial_Right	rTHAvm	Tha/HY
59	Hypothalamus_Lateral_Left	lHYl	Tha/HY
60	Hypothalamus_Lateral_Right	rHYl	Tha/HY
61	Hypothalamus_Medial_Left	lHYm	Tha/HY
62	Hypothalamus_Medial_Right	rHYm	Tha/HY
63	Bed_Nucleus_Stria_Terminalis_Left	lBNST	BF
64	Bed_Nucleus_Stria_Terminalis_Right	rBNST	BF
65	Septum_Left	lSEP	BF
66	Septum_Right	rSEP	BF
67	Substantia_Innominata_Left	lSI	BF
68	Substantia_Innominata_Right	rSI	BF
69	IPAC_Left	lIPAC	BF
70	IPAC_Right	rIPAC	BF
71	Medial_Geniculate_Left	lMG	Brainstem
72	Medial_Geniculate_Right	rMG	Brainstem
73	Medulla_Left	lMY	Brainstem
74	Medulla_Right	rMY	Brainstem
75	Mesencephalic_Region_Left	lMES	Brainstem
76	Mesencephalic_Region_Right	rMES	Brainstem
77	PAG_Left	lPAG	Brainstem
78	PAG_Right	rPAG	Brainstem
79	Pons_Left	lPon	Brainstem
80	Pons_Right	rPon	Brainstem
81	Raphe_Left	lRA	Brainstem
82	Raphe_Right	rRA	Brainstem
83	Substantia_Nigra_Left	lSN	Brainstem
84	Substantia_Nigra_Right	rSN	Brainstem
85	Superior_Colliculus_Left	lSC	Brainstem
86	Superior_Colliculus_Right	rSC	Brainstem
87	Ventral_TegmentalArea_Left	lVTA	Brainstem
88	Ventral_TegmentalArea_Right	rVTA	Brainstem

### 2.7 Statistical analysis

We applied permutation test (10,000 times) to test the differences between groups in topological and regional metrics. The false discovery rate (FDR) method was used to correct the multiple comparisons in topological and regional metrics. To complement the permutation-based statistical analysis, Hedges’ g was calculated to estimate the effect size of between-group differences. Hedges’ g is a bias-corrected standardized mean difference that is recommended for studies with relatively small sample size. For altered functional connectivity induced by UAC, the network-based statistics (NBS) approach was applied (Zalesky et al., 2010). Before NBS analysis, Fisher’s z-transformation was applied to the functional connectivity matrices to improve the normality of correlation coefficients. NBS controls for multiple comparisons across the large number of network connections and identifies connected subnetworks showing significant between-group differences. Subnetworks were considered significant at a corrected threshold of *p* < 0.05.

## 3 Results

### 3.1 Altered regional activity of the proprioceptive pathway in UAC rats

**Figure 1:**
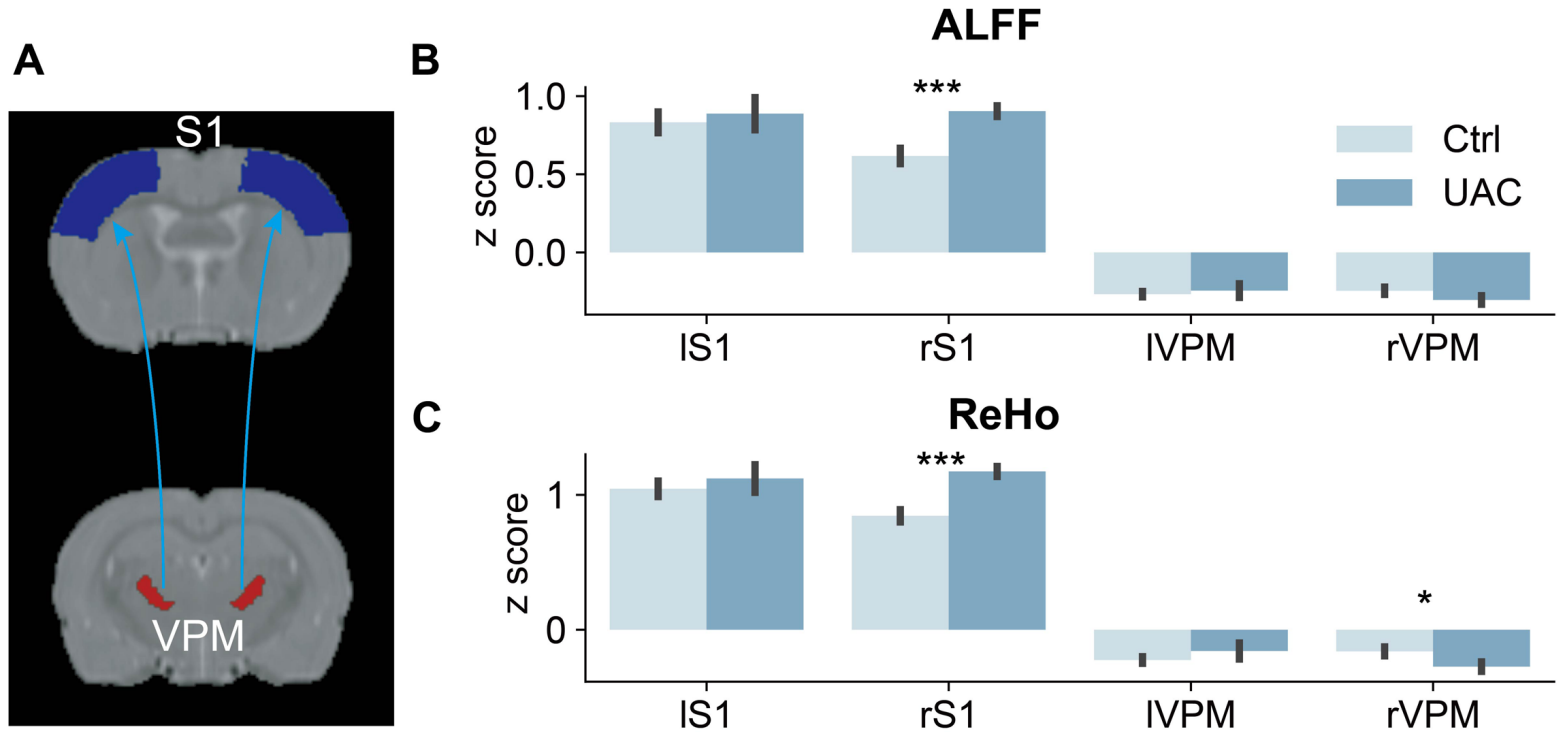
UAC modulates regional activity in the VPM–S1 circuit. **(A)** Regions of interest (ROIs) of the bilateral primary somatosensory cortex (S1) and ventral posteromedial thalamic nucleus (VPM). **(B)** Amplitude of low-frequency fluctuation (ALFF) in bilateral S1 and VPM. **(C)** Regional homogeneity (ReHo) in bilateral S1 and VPM. S1, primary somatosensory cortex; VPM, ventral posteromedial nucleus; l, left; r, right; UAC, unilateral anterior crossbite; Ctrl; control. *, *p* < 0.05; **, *p* < 0.01; ***, *p* < 0.001. Error bars represent the standard error of the mean.

First, we examined whether the regional activity of VPM and S1 in the proprioceptive pathway was modulated by the UAC (Figure 1A). Analysis of regional homogeneity (ReHo) and amplitude of low frequency fluctuation (ALFF) within these two regions revealed that both ALFF (*p* < 0.001, FDR corrected *p* < 0.05, Hedges’ *g* = 1.38) and ReHo (*p* < 0.001, FDR corrected *p* < 0.05, Hedges’ *g* = 1.62) in the right S1 was significantly increased in the UAC group compared with control animals. In contrast, no significant differences were found in the left S1 (*p* = 0.657 for ALFF, *p* = 0.513 for ReHo) (Figures 1B,C). For the VPM, ReHo in the right VPM was significantly reduced (*p* = 0.023, FDR corrected *p* < 0.05, Hedges’ *g* = −0.67) in UAC group, whereas ALFF in the right VPM showed no significant difference (*p* = 0.113). No significant differences were detected in the left VPM for either ALFF (*p* = 0.641) or ReHo (*p* = 0.303). We further tested whether UAC modulated the ipsilateral functional connectivity between S1 and VPM. Significantly increased ipsilateral VPM-S1 functional connectivity was observed in UAC rats compared with controls (left, *p* = 0.017, Hedges’ *g* = 0.64; right, *p* = 0.014, Hedges’ *g* = 0.65, see Supplementary Figure S1).

### 3.2 Network reconfiguration in UAC rats

**Figure 2:**
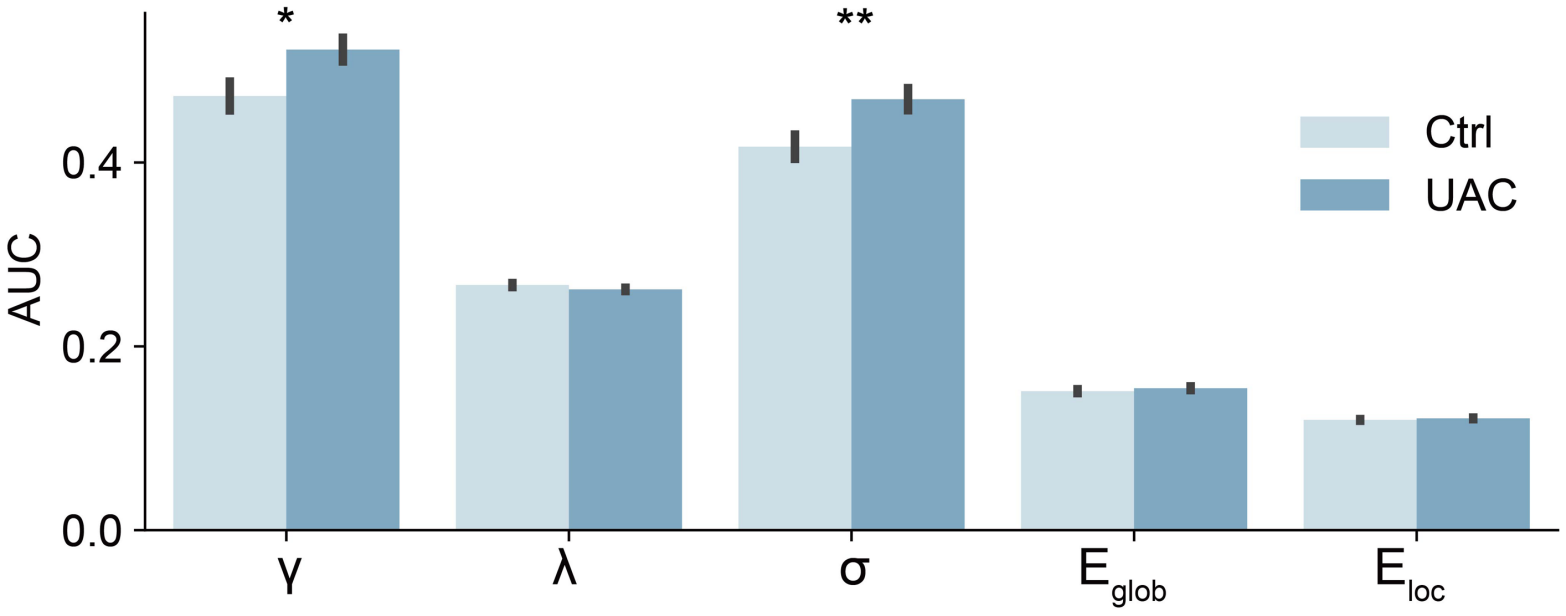
Alterations in the topology of functional brain networks in UAC rats. AUC, area under curve; *γ*, normalized clustering coefficient; *λ*, normalized characteristic path length; *σ*, small-worldness; E_glob_, global efficiency; E_loc_, local efficiency; UAC, unilateral anterior crossbite; Ctrl; control. *, *p* < 0.05; **, p < 0.01; ***, *p* < 0.001; 10,000 permutation tests. Error bars represent the standard error of the mean.

Next, we investigated the long-term effects of UAC on the topology of the functional brain networks in rats. Both groups exhibited small-world network properties (i.e., *γ* > 1, *λ* ≈ 1, and *σ* > 1) across the entire sparsity range (see Supplementary Figure S2). Higher normalized clustering coefficient (γ, *p* = 0.014, FDR corrected *p* < 0.05, Hedges’ *g* = 0.72) and small-worldness (σ, *p* = 0.006, FDR corrected *p* < 0.05, Hedges’ *g* = 0.83) were observed in UAC group compared with control group (Figure 2). In contrast, no significant differences were observed in normalized characteristic path length (λ, *p* = 0.109), global efficiency (*p* = 0.191), and local efficiency (*p* = 0.272) between the two groups (Figure 2).

**Figure 3:**
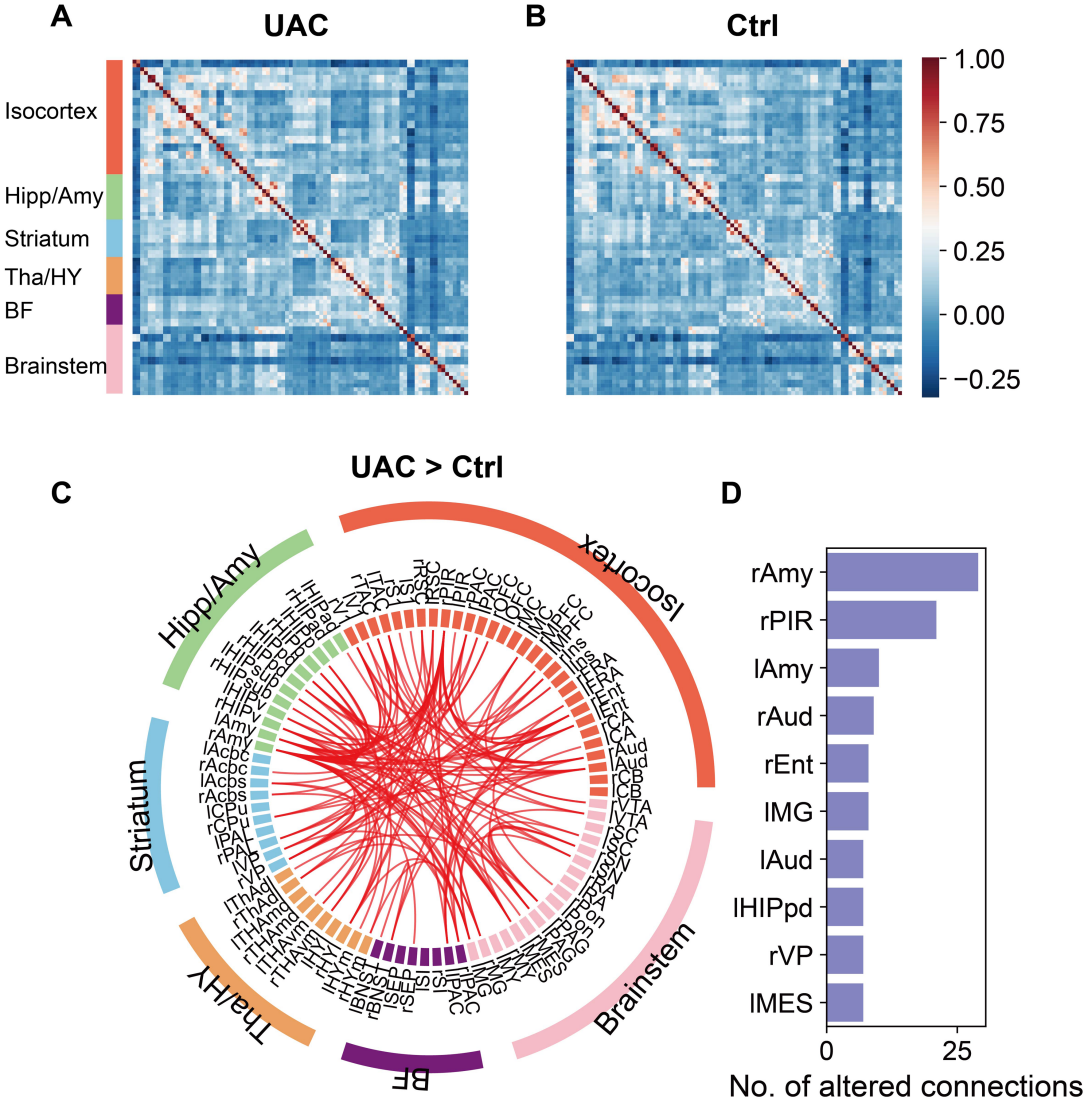
Altered functional connectivity networks in UAC rats. **(A,B)** Mean functional connectivity matrices for the UAC and control groups. The color bar represents Pearson’s correlation coefficients. **(C)** Subnetwork showing increased functional connectivity in the UAC group compared with the control group. **(D)** The top 10 brain regions ranked by the number of altered functional connections. UAC: Unilateral anterior crossbite; Ctrl: Control; Hipp: Hippocampus; Amy: Amygdala; Tha: Thalamus; HY: Hypothalamus; BF: Basal forebrain; Ceb: Cerebellum. See Table 1 for abbreviations of brain regions.

To further explore alterations in functional connectivity that might contribute to the observed network reconfiguration, we compared whole-brain functional connectivity between the UAC and control groups (Figures 3A,B). Network-based statistics revealed a single significantly increased subnetwork in the UAC group compared with control group (*p* = 0.023, NBS corrected, Hedges’ *g* = 0.86). This subnetwork consisted of 133 functional connections involving 66 of the 88 brain regions included in the whole-brain network (Figure 3C). We then counted the number of altered functional connectivity connected to each brain region and found that the right amygdala had the highest number of altered functional connections (Figure 3D). According to a previous study (Sporns et al., 2007), hub regions can be defined as nodes with a degree greater than the network mean plus one standard deviation. Within the identified subnetwork, the mean nodal degree was 4.0 and the standard deviation was 4.4, yielding a hub threshold of 8.4. The right amygdala with a degree of 29 is a hub region within the altered subnetwork.

**Figure 4:**
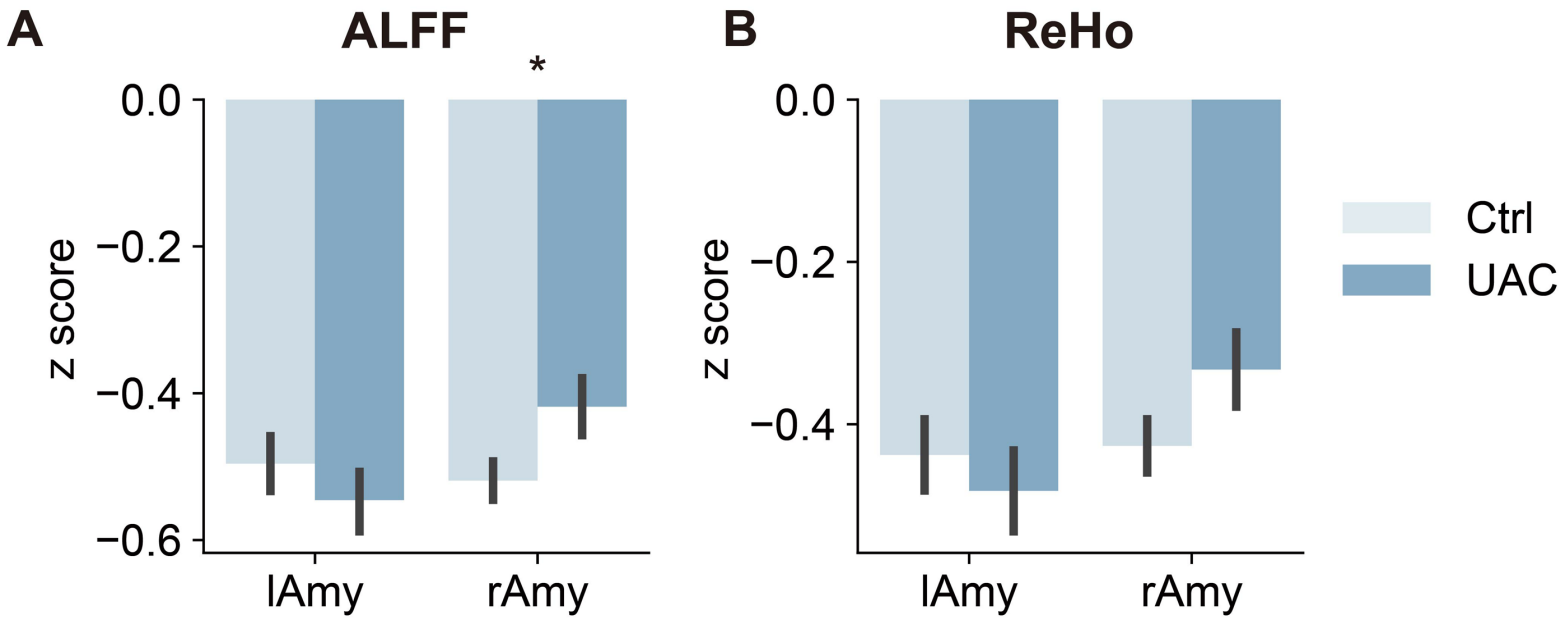
Altered regional activity of the amygdala following UAC. **(A)** Amplitude of low-frequency fluctuation (ALFF) in the left and right amygdala in the UAC and control groups. **(B)** Regional homogeneity (ReHo) in the left and right amygdala in the UAC and control groups. lAmy, left amygdala; rAmy, right amygdala; UAC, unilateral anterior crossbite; Ctrl, control. *, *p* < 0.05; **, *p* < 0.01; ***, *p* < 0.001. Error bars represent the standard error of the mean.

Finally, we checked the regional activity of bilateral amygdala after UAC (Figure 4). No significant differences were found in ALFF (*p* = 0.346) and ReHo (*p* = 0.502) in the left amygdala. In contrast, significantly higher ALFF (*p* = 0.039, Hedges’ *g* = 0.62) in the right amygdala was observed in UAC group than control group, whereas ReHo in the right amygdala showed no significant difference (*p* = 0.100).

## 4 Discussion

In the present study, we investigated brain network reorganization in rat UAC model. Our results, for the first time, demonstrated significant alterations in brain network topology in UAC rats. Specifically, enhanced functional connectivity was identified within a subnetwork that was strongly associated with the right amygdala. In addition, abnormal regional activity was detected in the right amygdala of UAC rats. Furthermore, we found that the classical proprioceptive pathway, particularly the VPM-S1 circuit, was modulated by UAC modeling, with more prominent effects observed in the right hemisphere contralateral to the UAC modeling side. It has been indicated that the brain networks in rodents and humans share conserved organizational properties. That allows findings from rodent models to provide translational insights into human brain function (Pagani et al., 2023). Our present data provide an explain for clinical mental complains, such as in patients with dental occlusion problems (Watanabe et al., 2025).

The brain typically exhibits a small-world network architecture that supports efficient local specialization and global information integration (Bullmore and Sporns, 2009). Although UAC rats showed increased small-worldness (*σ*), this alteration does not necessarily indicate a more optimal network organization. The increase in small-worldness was primarily driven by an elevated normalized clustering coefficient (*γ*), whereas the normalized characteristic path length (*λ*) remained unchanged. Additionally, neither global nor local efficiency differed significantly between groups, suggesting that overall information processing efficiency was not improved in UAC rats. In graph theory, γ reflects the clustering property of the empirical network relative to matched random networks (Rubinov and Sporns, 2010). Collectively, these graph-theoretical findings suggest that chronic malocclusion is associated with alterations of whole-brain functional networks toward greater local clustering relative to random topology while preserving the overall balance between local specialization and global integration.

The NBS analysis identified a subnetwork with significantly increased functional connectivity in UAC rats. Resting-state functional connectivity reflects the temporal synchronization of spontaneous neural activity between spatially distributed brain regions (Fox and Raichle, 2007). Accordingly, increased functional connectivity is widely interpreted as reflecting enhanced neural synchronization and, in several neurological and psychiatric disorders, pathological hypersynchronization (Uhlhaas and Singer, 2006). Therefore, the enhanced functional connectivity observed within the identified subnetwork may reflect pathological hypersynchronization of spontaneous brain activity associated with chronic malocclusion.

The Vme-Vpdm-VPM-S1 circuit is known to transmit proprioceptive information to the somatosensory cortex (Liu et al., 2017; Ji et al., 2022). In the present study, we focused on the macroscale thalamocortical sensory pathway and observed abnormal regional activity in the VPM and S1 regions contralateral to the modeling side. Here we argue that the abnormalities in the VPM and S1 are caused by proprioceptive dysfunction rather than pain. Our previous study showed that UAC rats exhibit short-term ipsilateral orofacial hyperalgesia, with the pressure pain threshold returning to baseline within 2 weeks (Jing et al., 2017). In our present study, the rs-fMRI data were acquired after 20 weeks of UAC, well beyond the period of transient orofacial hyperalgesia. These results therefore provide functional evidence that abnormal activity within the proprioceptive pathway in malocclusion is associated with altered proprioceptive signaling.

Previous studies consistently reported increased anxiety-like behaviors in the UAC rodent model (Liu et al., 2019; Ji et al., 2022). In the current study, we identified a hypersynchronized subnetwork in UAC rats in which the right amygdala served as a central hub. The amygdala plays a critical role in emotional processing and has been strongly implicated in the pathophysiology of anxiety (Etkin and Wager, 2007; Tye et al., 2011). Consistent with this finding, our regional analysis further revealed increased activity in the right amygdala of UAC rats. Neuroimaging studies have consistently reported amygdala dysfunction in patients with anxiety disorders (Mochcovitch et al., 2014; Makovac et al., 2016; Mizzi et al., 2022). Within anxiety-related neural circuits, the amygdala receives afferent inputs from multiple brain regions, including the hippocampus, mPFC, and locus coeruleus (LeDoux, 2007; Gong, 2025). It also sends projections to several areas, such as the BNST, hippocampus, anterior cingulate cortex, and hypothalamus. This interconnected circuit, centered on the amygdala, are crucial for coordinating behavioral and physiological responses to potential threats. In line with these anatomical connections, the amygdala emerged as a key region in our data-driven brain network analysis. Collectively, these findings highlight the involvement of the amygdala in UAC rats and suggest that the dysfunction of amygdala may contribute to the anxiety-like behaviors induced by UAC.

Several limitations should be considered when interpreting the findings of the present study. First, although our previous studies demonstrated increased anxiety-like behaviors in the UAC rodent model (Liu et al., 2019; Ji et al., 2022), behavioral assessments were not conducted in the present study due to accidental animal deaths during transportation. This limitation prevented further exploration of the direct relationship between neuroimaging abnormalities and behavioral outcomes. Future studies combining individual-level neuroimaging analysis with comprehensive behavioral testing will help clarify the neural mechanisms underlying malocclusion-related behavioral alterations. Second, the metal tubes used for UAC model induction had to be removed prior to MRI scanning, which may have introduced unknown effects on the imaging results. The development and application of MRI-compatible materials in future studies could improve methodological rigor. Third, the relatively small sample size may limit statistical power and the generalizability of the findings; therefore, larger cohorts are needed to further validate and extend these results. In addition, the absence of a baseline imaging session precludes within-subject comparisons and limits our ability to determine whether the observed group differences reflect changes induced by UAC or pre-existing inter-individual variability between groups, even though such possibilities were very low. Finally, direct manipulation of amygdala activity will be necessary to further clarify its specific role in UAC-induced anxiety.

## 5 Conclusion

In conclusion, rats subjected to UAC exhibited alterations in whole-brain functional network organization, with the contralateral amygdala acting as a central hub within a hypersynchronized subnetwork. These findings provide new insights into large-scale brain network adaptations following chronic abnormal orofacial input.

## Data Availability

The raw data supporting the conclusions of this article will be made available by the authors, without undue reservation.

## Footnotes

1
https://github.com/CoBrALab/RABIES

## References

[ref1] Achard S. Bullmore E. (2007). Efficiency and cost of economical brain functional networks. PLoS Comput. Biol. 3:e17. doi: 10.1371/journal.pcbi.0030017, 17274684 PMC1794324

[ref2] Alsulaiman O. A. Alghannam M. I. Almazroua D. M. Alamri A. S. Shahin S. Y. Nassar E. A. . (2025). Mental health and malocclusion: a comprehensive review. Clin Pract 15:44. doi: 10.3390/clinpract15030044, 40136580 PMC11941310

[ref3] Barriere D. A. Magalhaes R. Novais A. Marques P. Selingue E. Geffroy F. . (2019). The SIGMA rat brain templates and atlases for multimodal MRI data analysis and visualization. Nat. Commun. 10:5699. doi: 10.1038/s41467-019-13575-7, 31836716 PMC6911097

[ref4] Bassett D. S. Sporns O. (2017). Network neuroscience. Nat. Neurosci. 20, 353–364. doi: 10.1038/nn.4502, 28230844 PMC5485642

[ref5] Brynildsen J. K. Hsu L. M. Ross T. J. Stein E. A. Yang Y. Lu H. (2017). Physiological characterization of a robust survival rodent fMRI method. Magn. Reson. Imaging 35, 54–60. doi: 10.1016/j.mri.2016.08.010, 27580522

[ref6] Bullmore E. Sporns O. (2009). Complex brain networks: graph theoretical analysis of structural and functional systems. Nat. Rev. Neurosci. 10, 186–198. doi: 10.1038/nrn2575, 19190637

[ref7] Cenzato N. Nobili A. Maspero C. (2021). Prevalence of dental malocclusions in different geographical areas: scoping review. Dent J (Basel) 9:117. doi: 10.3390/dj9100117, 34677179 PMC8534899

[ref8] Chao-Gan Y. Yu-Feng Z. (2010). DPARSF: a MATLAB toolbox for "pipeline" data analysis of resting-state fMRI. Front. Syst. Neurosci. 4:13. doi: 10.3389/fnsys.2010.00013, 20577591 PMC2889691

[ref9] Chen X. Wang Z. Lv Q. Lv Q. van Wingen G. Fridgeirsson E. A. . (2022). Common and differential connectivity profiles of deep brain stimulation and capsulotomy in refractory obsessive-compulsive disorder. Mol. Psychiatry 27, 1020–1030. doi: 10.1038/s41380-021-01358-w, 34703025

[ref10] Desrosiers-Gregoire G. Devenyi G. A. Grandjean J. Chakravarty M. M. (2024). A standardized image processing and data quality platform for rodent fMRI. Nat. Commun. 15:6708. doi: 10.1038/s41467-024-50826-8, 39112455 PMC11306392

[ref11] Etkin A. Wager T. D. (2007). Functional neuroimaging of anxiety: a meta-analysis of emotional processing in PTSD, social anxiety disorder, and specific phobia. Am. J. Psychiatry 164, 1476–1488. doi: 10.1176/appi.ajp.2007.07030504, 17898336 PMC3318959

[ref12] Fox M. D. Raichle M. E. (2007). Spontaneous fluctuations in brain activity observed with functional magnetic resonance imaging. Nat. Rev. Neurosci. 8, 700–711. doi: 10.1038/nrn2201, 17704812

[ref13] Gong W. (2025). Research progress on the neural circuits mechanisms of anxiety. Front Neural Circuits 19:1609145. doi: 10.3389/fncir.2025.1609145, 40635883 PMC12238031

[ref14] Gozzi A. Zerbi V. (2023). Modeling brain Dysconnectivity in rodents. Biol. Psychiatry 93, 419–429. doi: 10.1016/j.biopsych.2022.09.008, 36517282

[ref15] Ji Y. Y. Liu X. Li X. Xiao Y. F. Ma T. Wang J. . (2022). Activation of the Vpdm(VGLUT1)-VPM pathway contributes to anxiety-like behaviors induced by malocclusion. Front. Cell. Neurosci. 16:995345. doi: 10.3389/fncel.2022.995345, 36605612 PMC9807610

[ref16] Jing L. Liu X. D. Yang H. X. Zhang M. Wang Y. Duan L. . (2017). ERK potentiates p38 in central sensitization induced by traumatic occlusion. Neuroscience 340, 445–454. doi: 10.1016/j.neuroscience.2016.11.012, 27865869

[ref17] LeDoux J. (2007). The amygdala. Curr. Biol. 17, R868–R874. doi: 10.1016/j.cub.2007.08.005, 17956742

[ref18] Liu X. Zhang C. Wang D. Zhang H. Liu X. Li J. . (2017). Proprioceptive mechanisms in occlusion-stimulated masseter hypercontraction. Eur. J. Oral Sci. 125, 127–134. doi: 10.1111/eos.12331, 28145597

[ref19] Liu X. Zhou K. X. Yin N. N. Zhang C. K. Shi M. H. Zhang H. Y. . (2019). Malocclusion generates anxiety-like behavior through a putative lateral Habenula-mesencephalic trigeminal nucleus pathway. Front. Mol. Neurosci. 12:174. doi: 10.3389/fnmol.2019.00174, 31427925 PMC6689965

[ref20] Lv Q. Yang L. Li G. Wang Z. Shen Z. Yu W. . (2016). Large-scale persistent network reconfiguration induced by ketamine in anesthetized monkeys: relevance to mood disorders. Biol. Psychiatry 79, 765–775. doi: 10.1016/j.biopsych.2015.02.028, 25837427

[ref21] Makovac E. Meeten F. Watson D. R. Herman A. Garfinkel S. N. H D. C. . (2016). Alterations in amygdala-prefrontal functional connectivity account for excessive worry and autonomic dysregulation in generalized anxiety disorder. Biol. Psychiatry 80, 786–795. doi: 10.1016/j.biopsych.2015.10.013 26682467

[ref22] Mizzi S. Pedersen M. Lorenzetti V. Heinrichs M. Labuschagne I. (2022). Resting-state neuroimaging in social anxiety disorder: a systematic review. Mol. Psychiatry 27, 164–179. doi: 10.1038/s41380-021-01154-6, 34035474

[ref23] Mochcovitch M. D. da Rocha Freire R. C. Garcia R. F. Nardi A. E. (2014). A systematic review of fMRI studies in generalized anxiety disorder: evaluating its neural and cognitive basis. J. Affect. Disord. 167, 336–342. doi: 10.1016/j.jad.2014.06.041, 25020268

[ref24] Ono Y. Yamamoto T. Kubo K. Y. Onozuka M. (2010). Occlusion and brain function: mastication as a prevention of cognitive dysfunction. J. Oral Rehabil. 37, 624–640. doi: 10.1111/j.1365-2842.2010.02079.x, 20236235

[ref25] Pagani M. Gutierrez-Barragan D. de Guzman A. E. Xu T. Gozzi A. (2023). Mapping and comparing fMRI connectivity networks across species. Commun. Biol. 6:1238. doi: 10.1038/s42003-023-05629-w, 38062107 PMC10703935

[ref26] Reis P. H. F. Laxe L. A. C. Lacerda-Santos R. Munchow E. A. (2022). Distribution of anxiety and depression among different subtypes of temporomandibular disorder: a systematic review and meta-analysis. J. Oral Rehabil. 49, 754–767. doi: 10.1111/joor.13331, 35398904

[ref27] Rubinov M. Sporns O. (2010). Complex network measures of brain connectivity: uses and interpretations. NeuroImage 52, 1059–1069. doi: 10.1016/j.neuroimage.2009.10.003, 19819337

[ref28] Schwarz A. J. Danckaert A. Reese T. Gozzi A. Paxinos G. Watson C. . (2006). A stereotaxic MRI template set for the rat brain with tissue class distribution maps and co-registered anatomical atlas: application to pharmacological MRI. NeuroImage 32, 538–550. doi: 10.1016/j.neuroimage.2006.04.214, 16784876

[ref29] Shrivastava M. Ye L. (2023). Neuroimaging and artificial intelligence for assessment of chronic painful temporomandibular disorders-a comprehensive review. Int. J. Oral Sci. 15:58. doi: 10.1038/s41368-023-00254-z, 38155153 PMC10754947

[ref30] Sporns O. Honey C. J. Kotter R. (2007). Identification and classification of hubs in brain networks. PLoS One 2:e1049. doi: 10.1371/journal.pone.0001049, 17940613 PMC2013941

[ref31] Tye K. M. Prakash R. Kim S. Y. Fenno L. E. Grosenick L. Zarabi H. . (2011). Amygdala circuitry mediating reversible and bidirectional control of anxiety. Nature 471, 358–362. doi: 10.1038/nature09820, 21389985 PMC3154022

[ref32] Uhlhaas P. J. Singer W. (2006). Neural synchrony in brain disorders: relevance for cognitive dysfunctions and pathophysiology. Neuron 52, 155–168. doi: 10.1016/j.neuron.2006.09.020, 17015233

[ref33] van den Heuvel M. P. Hulshoff Pol H. E. (2010). Exploring the brain network: a review on resting-state fMRI functional connectivity. Eur. Neuropsychopharmacol. 20, 519–534. doi: 10.1016/j.euroneuro.2010.03.008, 20471808

[ref34] Wan J. Lin J. Zha T. Ciruela F. Jiang S. Wu Z. . (2025). Temporomandibular disorders and mental health: shared etiologies and treatment approaches. J. Headache Pain 26:52. doi: 10.1186/s10194-025-01985-6, 40075300 PMC11899861

[ref35] Wang J. Wang X. Xia M. Liao X. Evans A. He Y. (2015). GRETNA: a graph theoretical network analysis toolbox for imaging connectomics. Front. Hum. Neurosci. 9:386. doi: 10.3389/fnhum.2015.00386, 26175682 PMC4485071

[ref36] Watanabe M. Takao C. Maeda C. Yonemitsu I. Kimura Y. Tominaga R. . (2025). Case report: challenges of orthodontic treatment in patients with autism spectrum disorders diagnosed in adulthood. Front. Psych. 16:1558789. doi: 10.3389/fpsyt.2025.1558789, 40821015 PMC12352318

[ref37] Watts D. J. Strogatz S. H. (1998). Collective dynamics of 'small-world' networks. Nature 393, 440–442. doi: 10.1038/30918, 9623998

[ref38] Yin Y. He S. Xu J. You W. Li Q. Long J. . (2020). The neuro-pathophysiology of temporomandibular disorders-related pain: a systematic review of structural and functional MRI studies. J. Headache Pain 21:78. doi: 10.1186/s10194-020-01131-4, 32560622 PMC7304152

[ref39] Yin D. Zhang Z. Wang Z. Zeljic K. Lv Q. Cai D. . (2019). Brain map of intrinsic functional flexibility in anesthetized monkeys and awake humans. Front. Neurosci. 13:174. doi: 10.3389/fnins.2019.00174, 30873000 PMC6403192

[ref40] Zalesky A. Fornito A. Bullmore E. T. (2010). Network-based statistic: identifying differences in brain networks. NeuroImage 53, 1197–1207. doi: 10.1016/j.neuroimage.2010.06.041, 20600983

[ref41] Zhang X. Dai J. Lu L. Zhang J. Zhang M. Wang Y. . (2013). Experimentally created unilateral anterior crossbite induces a degenerative ossification phenotype in mandibular condyle of growing Sprague-Dawley rats. J. Oral Rehabil. 40, 500–508. doi: 10.1111/joor.12072, 23675932

[ref42] Zhu H. Tao Y. Wang S. Zhu X. Lin K. Zheng N. . (2024). fMRI, LFP, and anatomical evidence for hierarchical nociceptive routing pathway between somatosensory and insular cortices. NeuroImage 289:120549. doi: 10.1016/j.neuroimage.2024.120549, 38382864

[ref43] Zou J. Meng M. Law C. S. Rao Y. Zhou X. (2018). Common dental diseases in children and malocclusion. Int. J. Oral Sci. 10:7. doi: 10.1038/s41368-018-0012-3, 29540669 PMC5944594

