# Supplementary material for: Functional brain network alterations in a rat model of dental malocclusion
Source: Front Neurosci. 2026 Jul 28;20:1873417. doi: 10.3389/fnins.2026.1873417 (PMC13457461; doi:10.3389/fnins.2026.1873417)
Supplement: Supplementary file 1 [file Data_Sheet_1.docx]

**Title Page**

**Functional brain network alterations in a rat model of dental malocclusion**

**Qian Lv^1^, Jiali Xu^2^, Yuejiao Zhang^2^, Shuang Chen^3^, Meiqing Wang^#2,4^**

^1^State Key Laboratory of Brain Function and Disorders and MOE Frontiers Center for Brain Science and the Institutes of Brain Science, Fudan University, Shanghai, China

^2^Department of Oral Anatomy and Physiology, Shanghai Stomatological Hospital and School of Stomatology, Fudan University, Shanghai, China

^3^Department of Prosthodontics, Shanghai Stomatological Hospital and School of Stomatology, Fudan University, Shanghai, China

^4^Department of Oral Anatomy and Physiology, College of Stomatology, the Fourth Military Medical University, Xi’an, China

**^#^Corresponding Author:**

Meiqing Wang: wangmq0609@163.com

**Running Title:**

Dental occlusion induced Brain Changes

**Keywords:**

functional MRI, dental malocclusion, anxiety, brain network, amygdala, orofacial proprioceptive pathway

**Figure S1. Ipsilateral functional connectivity between S1 and VPM in UAC and control rats.** S1, primary somatosensory cortex; VPM, ventral posteromedial nucleus; l, left; r, right; UAC, unilateral anterior crossbite; Ctrl; control. *, *P* < 0.05; **, *P* < 0.01; ***, *P* < 0.001. Error bars represent the standard error of the mean.

**Figure S2. Network properties between UAC and control groups.** Small-world properties were plotted as a function of sparsity. UAC, unilateral anterior crossbite; Ctrl; control. Error bars represent the standard error of the mean.
